# Low educational status correlates with a high incidence of mortality among hypertensive subjects from Northeast Rural China

**DOI:** 10.3389/fpubh.2022.951930

**Published:** 2022-08-25

**Authors:** Shasha Yu, Xiaofan Guo, GuangXiao Li, Hongmei Yang, Liqiang Zheng, Yingxian Sun

**Affiliations:** ^1^Department of Cardiology, First Hospital of China Medical University, Shenyang, China; ^2^Department of Clinical Epidemiology, Institute of Cardiovascular Diseases, First Hospital of China Medical University, Shenyang, China; ^3^Department of Clinical Epidemiology, Shengjing Hospital of China Medical University, Shenyang, China

**Keywords:** education, hypertension, mortality, cardiovascular, rural

## Abstract

**Objective:**

Cumulative evidence indicates that education plays a major role in predicting cardiovascular risk factors. In this study, we intend to examine the possible relationship between education status and mortality in a large general subject from rural China.

**Methods:**

Adult hypertensive subjects (*n* = 5,227, age = 57.22 ± 10.18 years; 49.1% men) were recruited from general population surveys (Northeast China Rural Cardiovascular Health Study). Their educational status was categorized into two groups as follows: (1) Low education (illiterate or lower than primary school) and (2) medium-high education (higher than primary school). Cardiometabolic comorbidities, related cardiovascular risk factors, and echocardiographic measurements were analyzed in both groups.

**Results:**

Less educated hypertensive subjects had significantly higher prevalence of obesity, diabetes, dyslipidemia, and left ventricular hypertrophy than medium-high educated hypertensive subjects. In the medium-high educated subjects, a significant increase in left ventricular ejection fraction and lower rate of antihypertensive medication was found. Cox proportional hazards analysis indicated that medium-high education was independently associated with all-cause mortality (hazard ratio = 0.76; 95% confidence interval, 0.58, 0.99; *P* = 0.043) and cardiovascular mortality (hazard ratio = 0.65; 95% confidence interval, 0.44, 0.96; *P* = 0.028).

**Conclusion:**

Education may act as the best predictor of all-cause and cardiovascular mortality in rural hypertensive subjects. This finding suggests that in rural areas, education is likely to represent a cardiovascular specific risk factor and should be evaluated in the strategies of hypertension.

## Introduction

With the development of economy in rural areas, cardiovascular risk factors, such as hypertension, dyslipidemia, diabetes, and obesity have become a major disease burden, and account for a large proportion of mortality worldwide (1). Until 2013, in rural Chinese areas, the prevalence of lipid abnormality was 47.8, 13.8, 25.7, and 30.7% for total cholesterol (TC), high-density lipoprotein cholesterol (HDL-C), low-density lipoprotein cholesterol (LDL-C), and triglyceride (TG), respectively and the prevalence of hypertension was 51.1% (53.9% for men and 48.7% for women) (2, 3). Large cohort studies have demonstrated that high blood pressure (BP), as the major remediable risk factor, increased the risk of heart failure, atrial fibrillation, chronic kidney disease, heart valve diseases, aortic syndromes, and dementia, in addition to coronary heart disease and stroke (4–8). Although frequently asymptomatic, hypertension is associated with the cardiovascular disease (CVD) causation and is the leading cause of death globally (9, 10). Clinical trials have demonstrated that there is a decrease in mortality with reduction in BP, which emphasizes the importance of hypertension prevention (8). Moreover, BP reduction effectively reduces the overall burden of CVDs, significantly lowering the incidence of myocardial infarction, congestive heart failure, and cerebrovascular disease (11).

In 2020, The American Heart Association (AHA), in conjunction with the National Institutes of Health (NIH), reported the most up-to-date statistics related to heart disease, stroke, and cardiovascular risk factors (12). They present the AHA's My Life Check—Life's Simple 7 which includes the following: be active, maintain a healthy weight, learn about cholesterol, no smoking or use smokeless tobacco, eat a heart-healthy diet, keep blood pressure healthy, and learn about blood sugar and diabetes mellitus (12). Except for those factors mentioned above, in recent years, socio-economic status (SES) has gradually gained more attention and has been considered as an important factor for the development of CVDs. Among them, education status has been proved to be a useful indicator of SES and important predictor of CVD, and total and cardiovascular mortality (13, 14). Rural subjects from China have unique SES characters for example, compared with urban citizens, rural residents have relatively lower annual income, poor medical support, and higher rate of chronic diseases (15–20). Among those characters mentioned previously, the typical one is that lower educational status.

It is important to improve cardiovascular risk prediction to effectively prevent CVDs. Previous studies designed to estimate the possible effect of education on cardiovascular risk in hypertensive subjects suggested that education might serve as the best predictor of cardiovascular risk (21). Nevertheless, these studies enrolled participants from urban or developed areas who had relatively higher educational status than their rural counterparts. Besides, no study has evaluated whether education was able to predict cardiovascular mortality or all-cause mortality in hypertensive participants. To reduce cardiovascular mortality in rural areas of China, we need to identify useful predictors and develop useful strategies to control it. Hence, this study was designed to evaluate the impact of education on cardiovascular events (CVEs), and cardiovascular and all-cause mortality in rural hypertensive subjects.

## Materials and methods

### Subjects and methods

The Northeast China Rural Cardiovascular Health Study (NCRCHS) is a community-based prospective cohort study carried out in rural areas of Northeast China. The design and inclusion criteria of the study have been described previously (22). The study was approved by the Ethics Committee of China Medical University (Shenyang, China AF-SDP-07-1, 0-01). In brief, a multi-stage, stratified, and random cluster sampling design was adopted. In the first stage, Dawa, Zhangwu, and Liaoyang countries were selected from the eastern, southern, and northern regions of Liaoning Province. In the second stage, one town was randomly selected from each county (total of 3 towns). In the third stage, 8–10 rural villages from each town were randomly selected. In total, 26 rural villages were included. All eligible permanent residents (aged ≥35 years) were invited from each village to participate in the study. Participants who were pregnant, had a malignant tumor, or had mental disorders were excluded (23). For each participant, detailed information was collected at baseline. In 2015 and 2017, participants were invited to attend a follow-up study. Of the 11,956 subjects, 1,256 participants were not included due to missing contact information, and 10,349 participants (86.6%) completed at least one follow-up visit. The median follow-up was 4.66 years. Written informed consent was obtained from all participants. Complete information on co-variables was required at the baseline visit for inclusion in the current analyses. In the present study, we enrolled hypertensive subjects. Therefore, 5,227 participants out of 10,349 were enrolled in the present study.

All hypertensive subjects were divided into two groups according to their educational status as follows: (1) ‘Low educational group' consisting of 2,925 subjects (60% females) with a mean age of 60.52 ± 9.62 years who were illiterates or with an educational status of lower than primary school. (2) ‘Medium-high educational group' consisting of 2,302 subjects (38.9% females) with a mean age of 53.02 ± 9.29 years with higher than primary school education level, including high school, college, or above.

### Measurements

For all subjects, clinical examination, including medical history, anthropometry, BP measurement, Doppler echocardiology, and fasting collection of blood were conducted for determining biological parameters. All this has been described in detail in previous studies (22, 24, 25). Weight and height were measured in participants with light weight clothing and without shoes. Waist circumference was measured at the umbilicus using a non-elastic tape. Body mass index (BMI, weight in kilograms divided by the square of height) and waist-to-hip ratio (WHR) were calculated. BP was assessed thrice in participants seated, after at least 5 min of rest using a standardized automatic electronic sphygmomanometer (HEM-907; Omron, Tokyo, Japan). Hypertension was defined as a systolic blood pressure (SBP) of ≥140 mmHg and/or a diastolic blood pressure (DBP) of ≥90 mmHg, and/or use of antihypertensive medications (11). Additionally, we evaluated the status of smoking and drinking. Fasting blood samples were collected in the morning from participants who fasted for at least 12 h. Fasting plasma glucose (FPG), TC, LDL-C, HDL-C, and TG were analyzed enzymatically.

### Echocardiographic examination

All subjects underwent an M-mode and B-mode echocardiographic examination according to standardized procedures previously described (26). Left ventricular mass (LVM) and left ventricular mass index (LVMI) were calculated using data from echocardiographic examination. LVM was normalized for body surface area. Left ventricular hypertrophy (LVH) was defined as LVM index ≥115 g/m^2^ in men and 99 g/m^2^ in women (27, 28).

### Adjudication of endpoints

The median follow-up was 4.66 years. The first participant was enrolled in January 2012, whereas the last follow-up date was December 2017. A series of irregular follow-up visits were conducted. The follow-up time was determined as the interval period from the date of randomization to the date of death, the date of last visit, or the last recorded clinical event of participants still alive, whichever occurred first (29). The primary outcome of this study was cause-specific mortality and cardiovascular cause mortality. An incident CVE was defined as a composite of new onset stroke or coronary heart disease (CHD) during the follow-up period. The specific incidences of stroke and CHD were also determined. For all participants reporting possible diagnoses or death, all available clinical information was collected, including medical records and death certificates. All materials were independently reviewed and adjudicated by the end-point assessment committee. Stroke was defined according to the WHO Multinational Monitoring of Trends and Determinants in Cardiovascular Disease (MONICA) criteria. CHD was defined as the diagnosis of hospitalized angina, hospitalized myocardial infarction, CHD death, or any revascularization procedure (30).

### Statistical analysis

Descriptive statistics were calculated for all the variables, including continuous variables reported as mean ± standard deviations (SD) and categorical variables reported as numbers and percentages. Cox proportional hazards models were used to identify the associations between educational status and mortality with hazard ratios (HRs) and 95% confidence intervals (CIs) calculated. The related confounders were selected based on previous studies and their possible associations with cardiovascular mortality and all-cause mortality in clinical practice (21, 31, 32). All statistical analyses were performed using SPSS version 17.0 software, and *P*-values < 0.05 were considered statistically significant.

## Results

All hypertensive subjects were sub-divided into two groups according to their educational status, namely low educational and medium-high educational groups. There were 2,925 subjects with low education (55.96%), whereas 2,302 with medium-high education (44.04%). The results revealed that hypertensive subjects with medium-high education had significantly younger age and lower proportion of females than those with low education (Table 1). Besides, subjects with higher education had relatively higher value of BMI and DBP, whereas lower value of SBP, TC, and LDL-C than subjects with low education. Unexpectedly, the prevalence of current smoking (38.0 vs. 32.9%, *P* < 0.001) and drinking (30.9 vs. 20.1%, *P* < 0.001) were significantly higher among medium-high education subjects compared with low education subjects. Participants with low education had relatively higher proportion of low annual income compared with medium-high education participants (≤5,000 CNY/year: 18.0 vs. 10.0%). Additionally, the rate of empty-nest subjects was significantly higher in the low education group than the high education group (48.2 vs. 32.2%, *P* < 0.001).

**Table 1:** Clinical characteristics in hypertensive subdivided at baseline according to educational status.

	**Low education**	**Medium-high education**	***P*-value**
	**(*n* = 2,925)**	**(*n* = 2,302)**	
Sex (female)	1,762 (60.2)	896 (38.9)	<0.001
Age (years)	60.52 ± 9.62	53.02 ± 9.29	<0.001
BMI (kg/m^2^)	25.43 ± 3.74	25.82 ± 3.45	<0.001
WHR (%)	0.87 ± 0.08	0.87 ± 0.07	0.406
SBP (mmHg)	161.04 ± 20.29	157.10 ± 18.56	<0.001
DBP (mmHg)	87.96 ± 11.45	90.11 ± 10.73	<0.001
FPG (mmol/L)	6.16 ± 1.82	6.13 ± 1.93	0.592
TC (mmol/L)	5.50 ± 1.17	5.34 ± 1.04	<0.001
TG (mmol/L)	1.82 ± 1.72	1.77 ± 1.54	0.259
HDL-C (mmol/L)	1.43 ± 0.40	1.43 ± 0.41	0.858
LDL-C (mmol/L)	3.14 ± 0.89	3.08 ± 0.84	0.011
Current smoking (%)	963(32.9)	875(38.0)	<0.001
Current drinking (%)	588(20.1)	712(30.9)	<0.001
Annual income (CNY/year)			<0.001
≤ 5,000	527 (18.0)	231 (10.0)	
5,000–20,000	1,749 (59.9)	1,211 (52.6)	
>20,000	646 (22.1)	859 (37.3)	
Empty-nest subjects (Yes)	1,410 (48.2)	742 (32.2)	<0.001

BMI, body mass index; WHR, waist-to-hip ratio; SBP, systolic blood pressure; DBP, diastolic blood pressure; MBP, mean blood pressure; FPG, fasting plasma glucose; TC, total cholesterol; TG, triglyceride; HDL-C, high-density lipoprotein cholesterol; LDL-C, low-density lipoprotein cholesterol.

Table 2 showed that participants with low educational status had significantly higher prevalence of diabetes (17.1 vs. 14.9%, *P* = 0.02), dyslipidemia (82.2 vs. 77.9%, *P* < 0.001), and LVH (21.8 vs. 12.4%, *P* < 0.001) than participants with medium-high educational status. The prevalence of antihypertensive treatment was significantly higher in low educational group compared with medium-high educational group (32.8 vs. 26.1%, *P* < 0.001). In contrast, participants with medium-high education had relatively higher prevalence of obesity (57.5 vs. 52.2%, *P* < 0.001) and higher value of left ventricular ejection fraction (62.75 ± 3.89 vs. 61.95 ± 4.23, *P* < 0.001) than participants with low educational status. In terms of the prevalence of abdominal obesity, MetS, and E/A ratio, no significant differences were observed between the low educational and medium-high educational groups.

**Table 2:** Prevalence of comorbidities and echocardiographic parameters in hypertensives subdivided according to educational status.

	**Low education**	**Medium-high education**	***P*-value**
Case no.	2,925	2,302	
Obesity (%)	1,519 (52.2)	1,320 (57.5)	<0.001
Abdominal obesity (%)	1,766 (60.7)	1,408 (61.4)	0.340
Diabetes (%)	500 (17.1)	344 (14.9)	0.02
Dyslipidemia (%)	2,405 (82.2)	1,793 (77.9)	<0.001
MetS (%)	1,573 (54.6)	1,189 (52.4)	0.065
LVH (%)	617 (21.8)	275 (12.4)	<0.001
LVEF (%)	61.95 ± 4.23	62.75 ± 3.89	<0.001
E/A	0.85 ± 0.32	1.19 ± 0.32	0.055
Antihypertension treatment (%)	941 (32.8)	587 (26.1)	<0.001

MetS, metabolic syndrome; LVMI, left ventricular mass/BSA; LVH, left ventricular hypertrophy; IHD, chronic ischemic heart disease; LVEF, left ventricular ejection fraction; E/A, peak early transmitral flow/peak late transmitral flow.

Table 3 exhibits the Cox proportional hazard analysis after adjusting for possible confounders. Data showed that 308 deaths occurred from all-cause during follow-up. Participants with low education had remarkably higher incidence of all-cause mortality than those with medium-high education (7.6 vs. 3.8%, *P* < 0.001). Similarly, the incidence of CVD deaths was significantly higher in low education groups compared with medium-high education groups (4.3 vs. 1.7%, *P* < 0.001). Compared with the low education group, medium-high education group was associated with lower all-cause mortality (hazard ratio: 0.76, 95%CI, 0.58, 0.99, *P* = 0.043). In addition, 164 deaths attributed to CVDs and medium-high education was effective predictor of CVD deaths (hazard ratio: 0.65, 95%CI, 0.44, 0.96, *P* = 0.028). We also retrieved fatal and non-fatal CVEs. There were 161 CHD and 301 stroke events. Compared with low education group, the incidence of CVEs (10.3 vs. 6.3%, *P* < 0.001), CHD events (4.0 vs. 1.9%, *P* < 0.001), and stroke events (6.7 vs. 4.5%, *P* < 0.001) were significantly lower in medium-high education group. However, after adjusting for possible confounders, educational status was neither effective predictor of CVEs nor CHD events/stroke events.

**Table 3:** Association Between education levels and mortality.

		**Number**	**Low education (*n* = 2,925)**	**Medium-high education (*n* = 2,302)**	***P*-value**
Incidence (%)	All deaths	308	221 (7.6)	87 (3.8)	<0.001
	CVD deaths	164	125 (4.3)	39 (1.7)	<0.001
	CVEs (fatal/non-fatal)	445	301 (10.3)	144 (6.3)	<0.001
	CHD events (fatal/non-fatal)	161	117 (4.0)	44 (1.9)	<0.001
	Stroke events (fatal/non-fatal)	301	197 (6.7)	104 (4.5)	<0.001
Hazard ratio (95%CI)	All-cause mortality	308	1.00 (Ref)	0.76 (0.58, 0.99)	0.043
	CVD mortality	164	1.00 (Ref)	0.65 (0.44, 0.96)	0.028
	CVEs (fatal/non-fatal)	445	1.00 (Ref)	0.84 (0.68, 1.04)	0.130
	CHD events (fatal/non-fatal)	161	1.00 (Ref)	1.09 (0.75, 1.58)	0.651
	Stroke events (fatal/non-fatal)	301	1.00 (Ref)	1.89 (0.84, 1.41)	0.539

Models adjusted for age, sex, nation, current smoking, current drinking, total cholesterol, systolic blood pressure, body mass index, fasting plasma glucose, hypertensive medication and chronic disease status. CI, confidence interval; CVEs, Cardiovascular events; CVD, cardiovascular disease; HDL-C, high-density lipoprotein cholesterol.

## Discussion

The present study, for the first time, confirmed the association between educational status and mortality in hypertensive subjects from rural areas of China. The major novel finding of this study was that diabetes, dyslipidemia, LVH, and antihypertensive medications were higher in hypertensive subjects with less education than those with medium-high education, whereas obesity was more likely to be seen among higher educational subjects. This finding indicated that education has close relationship with important cardiovascular risk factors. Besides, after adjusting for possible confounders, relatively higher educational status was associated with lower incidence of all-cause mortality and cardiovascular mortality among hypertensive subjects.

The China Hypertension Survey reported a high prevalence of hypertension (23.2%) and prehypertension (41.3%) in the general population of China (33). Besides, there were no significant differences in hypertension prevalence between urban and rural residents (23.4 vs. 23.1%, *P* = 0.819) (33). This suggested that hypertension was not only prevalent among the urban population, but also among the rural population. Hypertension, especially uncontrolled and untreated hypertension, was associated with increased risk of total and cardiovascular mortality among general hypertensive population (34, 35). However, the possible confounders of the hypertension related cardiovascular mortality and all-cause mortality remain unclear. Therefore, it is necessary to determine the possible confounders that might be relevant to all-cause mortality and cardiovascular mortality among rural hypertensive subjects. In the present study, we enrolled subjects aged ≥35 years and the mean age was 57.21 ± 10.78 years (from 35 to 92 years) at baseline. The nine-year compulsory education in China started in 1986. Earlier, in rural areas of China, only few subjects could afford education, especially women. Data from our study inferred that in total 56.0% of hypertensive subjects (66.3% of women; 45.3% for men; *P* < 0.001) had primary or below school education. This result was significantly higher than previous studies held in rural or urban areas of China (33, 36). One possible reason could be that the previous studies enrolled general population. Hence, the average age was lower in these samples, which infers that younger subjects who were more likely to have better educational status were recruited. The relationship between educational levels and hypertension has been well-proved. Previous studies indicated that hypertensive subjects with poorly controlled BP are represented by low education level (37, 38). To our knowledge, this is the first study intended to evaluate the association of low education with all-cause mortality and cardiovascular mortality in rural hypertensive participants. The novel finding of the present study was that all-cause mortality and cardiovascular mortality were higher in less educated subjects than medium-high educated hypertensive subjects. This finding was in line with the previous literature data, and indicated that education is an important predictor of cardiovascular mortality and all-cause mortality (39–41). Woodward et al. analyzed nearly 90,000 general subjects and claimed that those with a primary education had an increased risk of CVD, cardiovascular mortality, and all-cause mortality compared with individuals with a tertiary education (42). In contrast, higher education was associated with increased alcohol consumption and inversely related to smoking, BP, cholesterol levels, and diabetes (42). However, in our study, subjects with medium-high education showed a relatively higher rate of current drinking and smoking but lower cholesterol levels.

There might be some possible explanations that were relevant to this association between low educational status and cardiovascular or all-cause mortality. First, in our study, we identified that hypertensive subjects with low education status tended to have higher rate of low annual income that those with medium-high education (18.0 vs. 10.0%, *P* < 0.05). Besides, low education subjects were more likely to be empty-nest subjects (48.2 vs. 32.2%, *P* < 0.001). These results indicated that low education subjects were unable to meet the basic needs of life and were unlikely to get enough social support from their relatives. Second, our results indicated that the prevalence of diabetes, dyslipidemia, and LVH were relatively higher among low educational status than among medium-high educational status, which suggested that more cardiovascular risk factors are associated with low education status. It has been proved that individuals with low education tend to have an increased number of CVD risk factors (43). Hu et al. found that almost half of the increased risk of incident acute myocardial infarction in low education groups was explained by the traditional risk factors (44). Third, among the enrolled participants, subjects with low educational status were older, which might explain the higher incidence of all-cause mortality and cardiovascular mortality than those with medium-high educational status. Even with these estimations, the definite mechanisms underlying the remainder of the increased risk correlated with low education remains to be determined. In addition to the relationship between educational status and cardiovascular/all-cause mortality, it has been demonstrated that low educational subjects were more likely to be associated with cardiometabolic factors, particularly diabetes, dyslipidemia, lower LVEF, and LVH. This was in line with previous studies (45, 46). In low education participants, it is possible to detect strong markers of systolic function impairment, including a significant reduction in LVEF, and of the prevalence of LVH.

One interesting finding of our study was that the antihypertensive treatment rate was significantly higher in low education group than in medium-high education group. This is in contrast with previous studies claiming that low education might adversely influence self-seeking behavior or access to healthcare. Previous studies confirmed that among hypertensives with poor BP control, a considerable group is represented by low education (37, 38). Even though participants with low education had relatively higher rate of antihypertensive treatment, the SBP was still higher among this group than the medium-high education group. This finding had been proved by other previous study which also reported that persons with low levels of education were more frequently treated than hypertensive subjects with higher levels of education (21). One possibility for this phenomenon might be due to the more awareness of non-drug approaches to lowering BP. They were more willingly to decrease BP by modifying lifestyle factors but not drug therapy (47). However, the relationship between education level and drug treatment of hypertension was not clearly elucidated in the literature. As for the possible reasons of this controversy, it might be relevant to the medication adherence. Even though there was a higher rate of antihypertensive treatment in low education group, subjects with low education might have a relatively lower levels of adherence which are associated with worse BP control, and adverse outcomes (48, 49). There were validated objective (e.g., pharmacy fill, electronic monitoring) and subjective (e.g., self-report) measures for assessing medication adherence (50). However, in our present study, we did not collect data about medication adherence. Therefore, further study was warranted in order to confirm this possibility. Another interesting finding of our study was that the incidence of obesity was relatively low in the low education group. A meta-analysis found that study region, age groups, gender, and observation period could affect the relationship between education and overweight/obesity (51). In our study, the mean age in low education group was higher compared with medium-high education group. As the age increased, the rate of obesity decreased simultaneously. This might partially account for the low incidence of obesity in low education participants. Besides, Mosli et al. found that individuals who had the highest income bracket with low levels of education might have greater odds of obesity (52). This indicates that the synergistic effect of income and education might lead to changes in the relationship between education and obesity. In our study, low education participants had relatively lower annual income compared with medium-high education (annual income ≤5,000 CNY/year: 18.0 vs. 10%).

Results of the present study concluded that low educational status at baseline was associated with high incidence of mortality. Since the educational status at baseline cannot be altered, it seemed that there was no way to control this risk factor to reduce mortality. However, that was not the case. To better control all-cause mortality and cardiovascular mortality, we should understand that it is not the actual educational status that affected the health. In fact, it is the relevant health knowledge or healthy lifestyle derived from education that played an important role in all-cause mortality and cardiovascular mortality. We can use education intervention to make up for the low education status. A previous study aimed to evaluate the effectiveness of a health education intervention in improving hypertension knowledge, prevention, and self-care practices among older hypertensive residents. This study confirmed that community-based health education intervention targeting old adults can increase hypertensive knowledge, and improve prevention and self-care practices of hypertension (53). Therefore, even though we cannot change the baseline education status, we can restrict mortality through community educational intervention.

## Limitations

Inevitably, there are certain limitations to the present study. (1) The study only evaluated whether subjects had antihypertensive medication. It did not list specific drugs that were used in the study. (2) The recruited subjects were from one province of northeast China, therefore, the findings cannot be generalized to all subjects across the country. (3) The definitions of diabetes, dyslipidemia, and MetS were based on a single blood test which might have bias. (4) Although we had controlled for a range of common confounders, the possibility of unmeasured and residual confounding might have contributed to the risk of mortality, which might distort the associations observed in this study. (5) Data about medication, specific kinds of antihypertensive medication, and other related factors were not included in the present study which caused the inability to elucidate the possible reasons for relative higher rate of antihypertensive treatment in low education group compared to medium-high education group.

## Conclusion

Our data clearly indicates a definite association between low educational status and mortality, which emphasizes the importance of education. To some extent, this finding has a significant guiding effect on policy making, and this policy should ensure that people from rural areas with health problems are not deprived due to educational opportunities. More community-based health educational interventions are encouraged to better control cardiovascular risk and mortality.

## Data availability statement

The raw data supporting the conclusions of this article will be made available by the authors, without undue reservation.

## Ethics statement

The studies involving human participants were reviewed and approved by the Ethics Committee of China Medical University (Shenyang, China AF-SDP-07-1, 0-01). All procedures were performed in accordance with ethical standards. Written consent was obtained from all participants after they had been informed of the objectives, benefits, medical items and confidentiality agreement regarding their personal information. The patients/participants provided their written informed consent to participate in this study.

## Author contributions

SY contributed to the data collection, analysis, and interpretation. XG and HY contributed to data collection. GL and SY contributed to the data analysis. YS contributed to the study conception and design. All authors read and approved the final version of the manuscript.

## Funding

This study was supported by grants from the National Key Research and Development Program from the Ministry of Science and Technology of China (Project Grant # 2018 YFC 1312400 and Sub-project Grant # 2018 YFC 1312403) and the China Medical University Youth Backbone Program project funds (grant no. QGZ2018037).

## Conflict of interest

The authors declare that the research was conducted in the absence of any commercial or financial relationships that could be construed as a potential conflict of interest.

## Publisher's note

All claims expressed in this article are solely those of the authors and do not necessarily represent those of their affiliated organizations, or those of the publisher, the editors and the reviewers. Any product that may be evaluated in this article, or claim that may be made by its manufacturer, is not guaranteed or endorsed by the publisher.
